# Innovative Multilayer Electrospun Patches for the Slow Release of Natural Oily Extracts as Dressings to Boost Wound Healing

**DOI:** 10.3390/pharmaceutics16020159

**Published:** 2024-01-24

**Authors:** Noemi Fiaschini, Fiorella Carnevali, Stephen Andrew Van der Esch, Roberta Vitali, Mariateresa Mancuso, Maria Sulli, Gianfranco Diretto, Anna Negroni, Antonio Rinaldi

**Affiliations:** 1 Nanofaber S.r.l., 00123 Rome, Italy; noemi.fiaschini@nanofaber.com; 2 Italian National Agency for New Technologies, Energy and Sustainable Development (ENEA), Casaccia Research Center, 00123 Rome, Italy; fiorella.carnevali@enea.it (F.C.); savdesch@gmail.com (S.A.V.d.E.); roberta.vitali@enea.it (R.V.); mariateresa.mancuso@enea.it (M.M.); maria.sulli@enea.it (M.S.); gianfranco.diretto@enea.it (G.D.)

**Keywords:** electrospinning, membranes, nanostructured scaffolds, polycaprolactone (PCL), poly(ethylene oxide) (PEO), biocompatibility, oily extract, wound dressing

## Abstract

Electrospinning is an advanced manufacturing strategy used to create innovative medical devices from continuous nanoscale fibers that is endowed with tunable biological, chemical, and physical properties. Innovative medical patches manufactured entirely by electrospinning are discussed in this paper, using a specific plant-derived formulation “1 Primary Wound Dressing©” (1-PWD) as an active pharmaceutical ingredient (API). 1-PWD is composed of neem oil (Azadirachta indica A. Juss.) and the oily extracts of *Hypericum perforatum* (L.) flowers, according to the formulation patented by the ENEA of proven therapeutic efficacy as wound dressings. The goal of this work is to encapsulate this API and demonstrate that its slow release from an engineered electrospun patch can increase the therapeutic efficacy for wound healing. The prototyped patch is a three-layer core–shell membrane, with a core made of fibers from a 1-PWD-PEO blend, enveloped within two external layers made of medical-grade polycaprolactone (PCL), ensuring mechanical strength and integrity during manipulation. The system was characterized via electron microscopy (SEM) and chemical and contact angle tests. The encapsulation, release, and efficacy of the API were confirmed by FTIR and LC-HRMS and were validated via in vitro toxicology and scratch assays.

## 1. Introduction

Electrospinning is a versatile and scalable technique for generating ultrathin nanofibers for coatings and self-standing membranes, offering a facile technology to create continuous nanomaterials with variable biological, chemical, and physical properties [1,2,3,4]. Limiting our scope to solution electrospinning, the materials and process parameters can be controlled and optimized via the design of experiments to modify the diameter distribution and overall quality of the resulting fibers [5,6,7].

Electrospun non-woven scaffolds have useful properties for wound care applications and wound dressings [8,9,10] and can be easily functionalized for those purposes, e.g., to provide an optimally moist wound environment or deliver bioactive agents and active pharmaceutical ingredients (APIs) to drive the healing process. The fibrous microstructure of such scaffolds mimics the native extracellular matrix, allowing for selective mass transfer, such as gas exchange and the removal of wound exudates, thus providing an ideal microenvironment for cell adhesion, proliferation, and subsequent differentiation [11]. Also, electrospinning allows relatively easy incorporation of different drugs or growth factors into the dressing matrix, making it a promising drug delivery system [12].

Among the many material systems that can be processed by electrospinning, polycaprolactone (PCL) and polyethylene oxide (PEO) are some relevant options.

PCL is a synthetic polyester that is FDA-approved for medical applications and widely investigated for tissue engineering and drug delivery due to its biocompatibility, biodegradability, non-toxicity, and superior mechanical properties compared to natural polymers. However, for the given application of wound healing, the hydrophobicity of PCL may limit the absorption of wound exudate and interactions with cells necessary for regeneration [13]. PEO, instead, is a natural, biocompatible, water-soluble polymer, used extensively in food and pharma industries for the release of active agents upon its dissolution in an aqueous environment [14].

This paper explores the potential of combining PCL and PEO through electrospinning to obtain an innovative composite construct with desirable mechanical and microstructural properties for drug delivery in wound healing. In particular, the focus is on the incorporation of natural bioactive APIs into the electrospun fibers, which is a rather recent trend and is relevant to the transition to a green economy [15,16,17].

The utilization of electrospun fibers for wound dressing applications, containing natural bioactive substances, represents a relatively recent technological advancement. Several research studies published over the past five years on this topic highlight the versatility of electrospun materials for diverse therapeutic applications, in addition to tailorable chemical and physical properties [14,15,16,17,18,19]. Our methodology adopts the manufacturing protocols developed by Nanofaber srl to produce oily extract-loaded electrospun membranes for wound healing applications. The specific API used in this study is a plant-derived formulation commercially known as “1 Primary Wound Dressing©” (1-PWD, hereafter), composed of neem oil (Azadirachta indica A. Juss.) and the oily extract of *Hypericum perforatum* (L.) flowers. 1-PWD is a class 2b medical device that has been authorized for topical use in humans since 2010 and from one month of age (pediatric use) since 2018 [20]. This API was formulated and patented by the ENEA following the extensive proof of its therapeutic efficacy in the wound healing process [21]. The anti-inflammatory, antimicrobial, and antifungal activities of 1-PWD have been widely demonstrated both in vitro and in vivo, and its safety on the wound bed in vivo has been priorly established [22]. Yet, 1-PWD has never been used in electrospinning.

In this context, this study demonstrates the manufacturing of a layered electrospun patch made of electrospun nanofibers loaded with 1-PWD and the enabling of its slow release. The final goal of this work is indeed the prototyping of a biodegradable biomedical patch that not only retains the therapeutic properties of 1-PWD but also ensures its gradual release for wound dressing purposes. Such an electrospun construct was characterized extensively to assess both physical properties (e.g., microstructure, average fiber diameter, porosity, wettability, etc.) and biological performance (e.g., cytotoxicity, rate of wound closure). The release rate of active principles from the patch was confirmed by LC-HRMS.

## 2. Materials and Methods

### 2.1. Materials

Polycaprolactone (PCL, MW = 80,000) was purchased from Perstorp (Lund, Sweden), and PEO or poly(ethylene glycol) with an Mw of 35,000 was provided by Fluka (Buchs, Switzerland). Chloroform and Dimethylformamide were obtained by VWR (Radnor, PA, USA).

The patented plant-derived formulation 1 Primary Wound Dressing© (1-PWD), composed of neem oil (Azadirachta indica A. Juss.) and the oily extract of *Hypericum perforatum* (L.) flowers, was supplied by the ENEA (Italian National Agency for New Technologies, Energy, and Sustainable Development).

### 2.2. Cell Lines

HT29 (HTB38, CL.19A) (human colorectal adenocarcinoma cell line) and NIH3T3 embryonic mouse fibroblast cells (ATCC number CRL-1658) were purchased from American Type Culture Collection (ATCC, Rockville, MA, USA).

HT29 was maintained at 37 °C and 5% CO_2_ in McCOY’s 5A medium (Gibco, Grand Island, NY, USA) supplemented with 10% inactivated fetal bovine serum (FBS Eu Approved, Euroclone, Milan, Italy), 2 mM L-Glutamine, 100 U/mL penicillin, and 100 μg/mL streptomycin (Euroclone).

NIH3T3 was grown in DMEM supplemented with 2 mm l-glutamine, 10% fetal bovine serum, and 1% penicillin/streptomycin (Euroclone) at a temperature of 37 °C in a humidified incubator with a 5% CO_2_ atmosphere.

### 2.3. Solutions Preparation and Electrospinning Process

A PCL solution was prepared from medical-grade PCL granules (CAPA^®^ 6800, average 80,000 MW, Perstorp, Sweden) dissolved at a 12% *w*/*v* concentration in a 65:35 solvent mixture of Chloroform (99.2% purity, stabilized with 0.6% ethanol, VWR, Radnor, PA, USA) and Dimethylformamide (100% purity, VWR, Radnor, PA, USA) under mild stirring overnight at room temperature. The supplier reported a theoretical density for the PCL of ρ0 = 1.145 g/cm^3^.

A PEO solution was prepared from PEO granules (35,000, FLUKA Chemicals, Buchs, Switzerland) dissolved at a 30% *w*/*v* concentration in distilled water under mild stirring at 50 °C for two days. The supplier reported a theoretical density for the PEO of ρ0 = 0.45 g/cm^3^.

1-PWD formulation, obtained by API spray dispensing directly from its container, was then mixed with the PEO solution (30 wt%) under magnetic stirring until the incorporation of a 4% product.

Biomedical scaffolds were fabricated with electrospinning using a pilot scale electrospinning station (Fluidnatek LE100, Bioinicia, Valencia, Spain) using different variations of a process recipe, provided in-kind by the company Nanofaber srl (Rome, Italy), varying the applied voltage and flow rate (FR). The relative humidity (RH) and ambient temperature during the deposition process were recorded.

### 2.4. Morphology Characterization of Membranes

The morphological properties of electrospun materials were examined using a field emission gun scanning electron microscope Leo 1530 model (ZEISS, Jena, Germany) without metal coating working at low voltage (i.e., 2 kV) to avoid charging effects and damage to the dielectric polymer from overheating.

### 2.5. Chemical Characterization of Membranes

The electrospun membranes were characterized by ATR-FTIR spectroscopy. The spectra were collected using a Nicolet iS50 spectrometer (Thermo Fisher Scientific, Waltham, MA, USA) equipped with an ATR accessory. The measurements were recorded using a diamond crystal cell ATR, typically using 32 scans at a resolution of 4 cm^−1^. The samples were all measured under the same mechanical force pushing the samples in contact with the diamond crystal. No ATR correction has been applied to the data.

### 2.6. Wettability Test and Contact Angles Measurements

The wettability tests of the membranes were conducted by measuring the contact angle using a Leica Wild M3Z stereomicroscope. Images were captured using a Moticam 2000. For the contact angle analysis, two representative liquids were used: deionized water (WCA) to represent the hydrophilic part and 1-Primary Wound Dressing© (1-PWD) to represent the lipophilic part (OCA). The measurement of the contact angle between the membrane and a droplet of the liquid was performed 10 s after depositing 10 μL of the liquid onto the membrane surface. Non-standard tests were performed to compare the wetting area of a droplet of a given size, either of water or 1-PWD, dispensed on a surface and observed in top view after 60 s of setting time.

### 2.7. In Vitro Oily Extract Release Analysis by LC/HESI/MS

A total of 0.2 mL of culture medium were extracted after 24 h/48 h/72 h of incubation with a 1-PWD patch with 0.5 mL 75% *v*/*v* MetOH/0.1% *v*/*v* formic acid, spiked with 0.5 µg/mL Formononetin (Sigma-Aldrich, St. Louis, MO, USA) as an internal standard. Samples were extracted at room temperature by continuous agitation for 30 min in MixerMill at 30 Hz and centrifuged at 20,000× *g* for 20 min; the supernatant was collected, filtered with HPLC PTFE filter tubes (0.2 µm pore size), and 5 µL were injected to the LC-MS system using a Ultimate HPLC (Dionex, Thermo Fisher Scientific, Waltham, MA, USA) coupled to a Q-Exactive Hybrid Quadrupole-Orbitrap Mass Spectrometer (Thermo Fisher Scientific, Waltham, MA, USA). LC was performed using the C18 Luna reverse-phase column (100 × 2.1 mm^2^, 2.5 µm; Phenomenex, Torrance, CA, USA), and LC settings and the solvent system were as previously reported [17]. The FTMS *m*/*z* range was 110–1600 *m*/*z*, the capillary temperature was 250 °C, the spray voltage was set to 3.5 kV, the probe heater temperature was 330 °C, and the S-lens RF level was set at 50. All the chemicals and solvents used during the entire procedure were of MS grade (Chromasolv, Merck Millipore, Darmstadt, Germany). Compounds were identified based on accurate masses in full MS (Δppm < 4) compared with available spectra using PubChem (https://pubchem.ncbi.nlm.nih.gov/) and MassBank (https://massbank.eu/MassBank/) databases. Relative values for detected metabolites are expressed as fold on the level of the internal standard (Fold-IS). Data are present as average ± standard deviation of 3 replicates (3 different patches, incubation and extraction procedures of culture medium incubated with oily extract-loaded electrospun PLC-PEO patches).

### 2.8. Viability Assay

Cell viability was evaluated using the MTT assay, employing 3-(4,5-dimethylthiazol-2-yl)-2,5-diphenyltetrazolium bromide (Sigma-Aldrich). Following a 24 and 72 h exposure period, the culture medium was removed, and 200 μL of MTT-containing medium (0.5 mg/mL) was added to the wells. Subsequently, the cells were incubated at 37 °C for 2 h. After incubation, the medium was discarded, and formazan crystals formed were dissolved using 200 μL of an SDS-DMF buffer (pH 4.7), followed by incubation at room temperature on an orbital shaker shielded from light for 35 min. Absorbance was then measured according to the Manufacturer’s instructions.

### 2.9. Fluorescent Stainings to Analyse Cell

Cells grown on electrospun membranes were fixed in formalin 4% in PBS for 10 min. Membrane diskettes placed at the bottom of a well plate were anchored in place by medical-grade polycarbonate c-rings provided by Nanofaber (Rome, Italy). Cells were washed in PBS, permeabilized by incubation in PBS 0.1% Triton X-100 (Sigma-Aldrich, St. Louis, MO, USA) for 10 min, and then blocked in PBS 1% BSA for 30 min. F-actin was stained with Alexa Fluor 488-conjugated Phalloidin (1:50, Molecular Probes, Eugene, OR, USA) according to the manufacturer’s instructions. 4′,6-diamidino-2-phenylindole (DAPI) was added for nuclei counterstaining. Images were acquired at a magnification of 4× with a Zeiss Axio Observer inverted fluorescent microscope using FITC and DAPI filters.

### 2.10. Wound Healing Assay (or Scratch Test)

A scratch test was performed using a Wound Healing Assay Kit (ab242285, Abcam, Cambridge, United Kingdom) according to the manufacturer’s instructions. The electrospun membrane disks were cut out by die punching to fit into the bottom of the 24-well plate. HT29 and NIH3T3 cells were cultured on electrospun patches in 24-well plates, respectively, at a density of 2.5 × 10^5^ and 3 × 10^5^ cells for 500 μL until confluence reached 90%. A straight-line wound was made using a “wound field” insert (cat. CBA-120, Cell BiolabsBIOLABS, Inc., Cambridge, United Kingdom), which ensured the anchoring at the well bottom of the patch disk. Cell debris from the wound field was removed by washing with PBS. The cells that migrated into the wounded area were visualized at 0, 24, and 48 h. Next, the cells on electrospun membranes were fixed in formalin 4% in PBS for 10 min, washed in PBS, permeabilized by incubation in PBS 0.1% Triton X-100 (Sigma) for 10 min, and finally blocked in PBS 1% BSA for 30 min. F-actin was stained with Alexa Fluor 488-conjugated Phalloidin (1:50, Molecular Probes) according to manufacturer’s instructions. 4′,6-diamidino-2-phenylindole (DAPI) was added for nuclei counterstaining. Images of each condition were acquired at a magnification of 4X with a Zeiss Axio Observer inverted fluorescent microscope using FITC and DAPI filters. Cellular density related to a fixed wounded area (1 mm^2^) was measured after 48 h using ImageJ software 1.53i (available in the public domain at www.nih.gov; National Institutes of Health (NIH), Bethesda, MD, USA) (10 acquisitions for each experimental point). The experiment was replicated three times.

### 2.11. Statistical Analysis

The original data were analyzed using ImageJ software, and then the data were subjected to a two-way ANOVA test. Data are expressed as the mean ± standard deviation (SD). *p*-values < 0.05 were considered to be statistically significant.

## 3. Results and Discussion

### 3.1. Membrane Production

**Figure 1:**
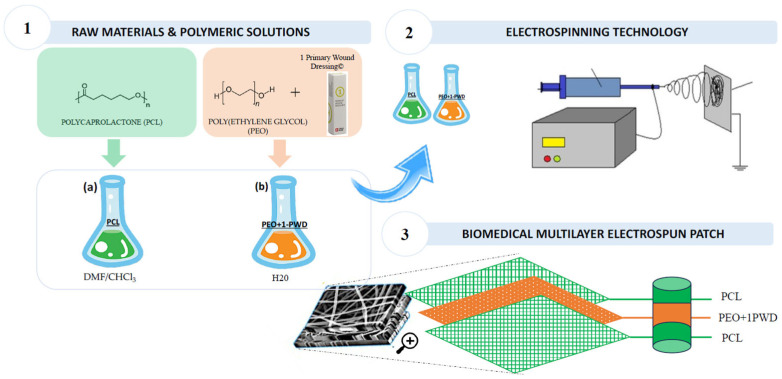
The experimental design of the functional nanofiber patch. Step 1: Granules of raw materials (polycaprolactone (PCL) and poly(ethylene glycole) (PEO)) used to prepare polymeric solutions using DMF/CHCL_3_ (for a solution) and H20 (for b solution); Step 2: Electrospinning process to obtain nanofiber membranes; Step 3: Biomedical multilayer obtained electrospun patch.

The three-layer composite wound dressing (hereafter referred to as the “patch”), schematically reported in Figure 1 was manufactured by electrospinning to reduce scar tissue formation while supporting proper wound healing. The top and bottom layers are thin electrospun layers of PCL nanofibers, providing membrane integrity and mechanical strength. Instead, the core layer is made of compound electrospun nanofibers, obtained from a mix of PEO and oily extracts from the selected API. The system was characterized via electron microscopy and chemical and contact angle tests. The encapsulation, release, and efficacy of the API were confirmed by HPLC. The biological performance was assessed by an in vitro scratch test.

### 3.2. Membrane Characterization

Membranes were characterized morphologically by scanning electron microscopy (SEM) and chemically by ATR Fourier-transform infrared spectroscopy (FTIR). The hydrophilicity of different layers of the membrane was assessed by water contact angle measurements.

#### 3.2.1. Morphological Analysis

**Figure 2:**
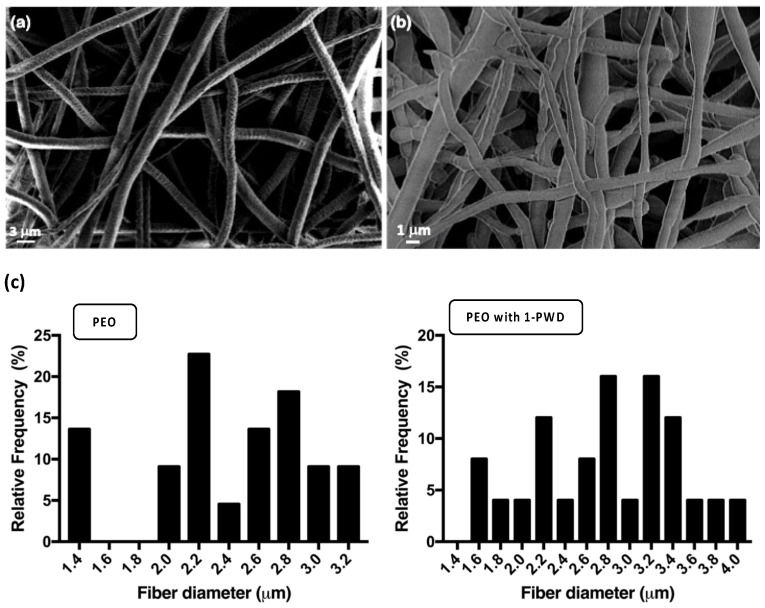
SEM micrographs at different magnifications of electrospun samples: (**a**) 4.5 K× of PEO and (**b**) 7.5 K× of PEO with 1-PWD. (**c**) The corresponding diameter distribution of different types of microfibers.

SEM images (Figure 2a,b) showed that the nanostructures of nanofiber mats were distributed randomly and uniformly with smooth fiber surfaces, showing a large surface area and a distinct porous structure. The corresponding fiber diameters for PEO alone and 1-PWD loaded with PEO were 2.40 ± 0.49 and 2.73 ± 0.74 μm, respectively (Figure 2c).

This change in fiber diameter is expected, as it is well-established that achieving a uniform, homogeneous, and smooth nanofiber morphology, free from beads, relies heavily on various electrospinning parameters and results in a given formulation that can be displaced by small variations in even one parameter, such as the inclusion of an API. Key parameters include polymer concentration, solution viscosity, electrical conductivity, temperature, and humidity. Specifically, a lower electrical conductivity of the solution can impact the elongation of the jet, resulting in larger fiber diameters. The observed increment in the PEO/1-PWD nanofiber diameter can, in this case, be likely attributed to the reduction in solution electrical conductivity, a phenomenon associated with the interaction between 1-PWD and PEO. Furthermore, an increase in solution viscosity is known to correlate with an augmented average fiber diameter. In our study, the addition of 1-PWD appears to have a reducing effect on solution viscosity due to its interaction with PEO. These findings align with similar results reported by Mori et al. [23], where the addition of candeia essential oil in various concentrations led to an increase in the average fiber diameter of PLA nanostructured mats. This emphasizes the importance of understanding and controlling factors influencing fiber diameter, as they directly impact the overall nanofiber morphology and, consequently, the performance of the material.

#### 3.2.2. Chemical Structure of Membranes

**Figure 3:**
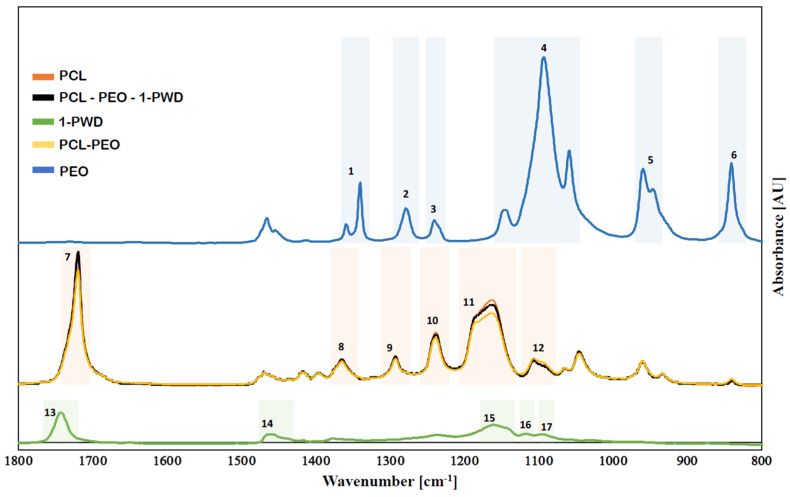
FTIR spectra; the number 1–17 indicate the characteristic peaks of different samples.

The chemical characteristics of different nanofiber mats were evaluated by Fourier-transform infrared spectroscopy (FTIR). The FTIR spectra of individual electrospun membranes of PCL. PEO and PEO-PCL, along with the one for pure 1-PWD, were compared to the spectrum obtained from the final multilayer system (Figure 3). The FTIR spectrum of PCL (Figure 3—peaks 7–12) exhibits distinct stretching and bending signals characteristic of the polymer. Within Band 7, at 1726 cm^−1^, there is a stretching vibration of the –C=O group. Additionally, asymmetric and symmetric stretching vibrations of C–O–C can be observed at 1242 and 1186 cm^−1^, stretching vibrations of the C–O and C–C groups in the crystalline and amorphous phases at 1295 and 1167 cm^−1^, and deformation vibrations of the –CH_2_ groups at 1370 cm^−1^ [24]. Figure 3 (peaks 1–6) shows the FITR spectrum of PEO. The peaks at 1467, 1361, 1276, and 839 cm^−1^ are assigned to C–H stretching, CH_2_ scissoring, CH_2_ wagging, CH_2_ twisting, and CH_2_ rocking. The peaks at 1143, 1091, and 1057 cm^−1^ are assigned to C–O–C stretching vibrations. The semi-crystalline phase of PEO is confirmed by the presence of a triple peak at C-O-C stretching [25]. The FTIR spectrum of 1-PWD (Figure 3—peaks 13–17), the API composed of neem oil, and the oily extract of Hypericum perforatum show the presence of some significant bands, such as 1463, 1234, 1161, 1118, and 1094 cm^−1^, indicating C=O stretching and C–H bending [16].

Results obtained from FTIR-ATR analyses revealed that 1-PWD oil was successfully loaded into the multilayer system, and compounds integrated with the structure did not result in any change in the chemical structure due to the interactions with the solvent.

#### 3.2.3. Hydrophilicity

**Figure 4:**
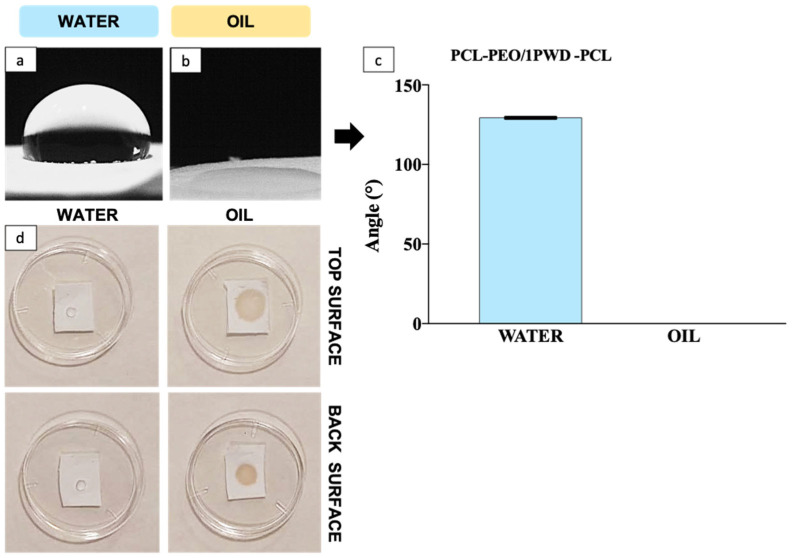
(**a**) Wet angle with polar liquid (water) on PCL after 10 s; (**b**) wet angle with apolar liquid (1-PWD) on PCL after 10 s; (**c**) contact angles of PCL-PEO/1-PWD-PCL (mean ± standard deviation); (**d**) qualitative comparison of pure PCL membranes vs. water and 1-PWD oil, showing the clear affinity between PCL and 1-PWD, as well as a difference between top and bottom surface on through-thickness imbibition extent.

Hydrophobicity is an important function for a wound dressing, preventing ingress of water from outside, which may cause infection and inflammation [26]. Hydrophilicity tests on PCL-PEO nanofiber mats were performed by contact angle measurement both for polar (i.e., water herein) and apolar (i.e., 1-PWD oil herein). The results in Figure 4a,b demonstrate how the multi-layer membrane is highly affine to oil and exhibits contact angle values much lower than water (i.e., 0° vs. 129.31 ± 0.81). The hydrophilicity of the PCL outer layers is markedly opposite to the strong affinity for the oil, where a conventional 0° value was assumed for the contact angle, given that the oil droplet was fully absorbed in the patch before the 10 s time interval foreseen in the method. A qualitative, yet telling, confirmation of the affinity of the PCL layer for the oil is provided by the test in Figure 4c, showing the extent of the same-sized droplet of water or oil after a set time of 60 s. The results display the intrinsic hydrophobic behavior of the electrospun PCL, which is identical on either side, whereas there is a clear through thickness and in-place adsorption in the case of 1-PWD, albeit with a larger wetting area in the case of dispensing onto the top surface (i.e., the surface being produced last in the electrospinning). The latter finding reveals that the PCL layer has some through-thickness microstructural gradients in fiber and pore distribution that cause a more efficient in-plane diffusion when the oil is fed from the top side. This can be advantageous for the design of a re-fillable patch, where the API can be reintroduced to replenish the medical device by dispending 1-PWD from the more efficient surface.

### 3.3. Biological Tests

Biological tests were aimed to ascertain membrane biocompatibility, cell viability, and proliferation rate and their ability to favor wound closure by a dedicated assay.

#### 3.3.1. Cell Viability and Biocompatibility

**Figure 5:**
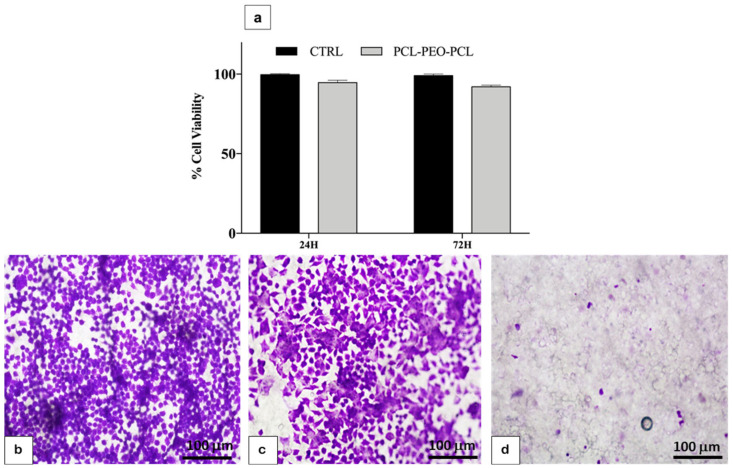
(**a**) Cell viability test (MTT); (**b**) HT29 cells on an electrospun membrane; (**c**) HT29 cells on an electrospun 1-PWD-loaded membrane; (**d**) HT29 cells on a membrane with 1-PWD sprayed by a commercial dispenser. Magnification: 10×. Scale bar = 100 μm.

Cell compatibility and viability of HT29, a human adenocarcinoma cell line with characteristics of epithelial cells, on membranes were investigated in pilot experiments. An MTT test was performed to assess membrane toxicity. Cells were seeded in standard wells without membrane and on the membrane alone, and an MTT assay was performed after 24 and 72 h. No significant toxic effects were shown (Figure 5a).

To evaluate which was the best method to incorporate 1-PWD in the electrospun membrane to obtain viable cells, three types of membranes were analyzed: one membrane produced without electrospun 1-PWD, one membrane with electrospun 1-PWD, and one membrane on which, after its production, 1-PWD was placed by directly spraying it from a commercial dispenser. Cells were seeded on the three kinds of membranes. Circle-shaped membranes were placed in 24-well dishes for cell cultures and wetted with a drop of medium for capillarity. A total of 50 μL of cell suspensions containing 2.5 × 10^5^ cells were added onto each membrane and incubated for 30 min (37 °C, 5% CO_2_) to maintain cell attachment before adding cell culture medium. After this incubation period, 200 μL of cell culture medium was added to each well. After 24 h of culture in an incubator at 37 °C and 5% CO_2_ conditions, membrane dishes were transferred on glass slides, fixed, stained with crystal violet, and observed with an optic microscope.

On membranes alone and 1-PWD, electrospun-loaded membrane cells exhibited phenotypical cell morphology and were firmly attached and well-spread on the well plate (Figure 5b,c). Membranes treated later with 1-PWD directly from their dispenser did not show viable cells on their surface (Figure 5d). Results demonstrated that 1-PWD electrospun-loaded membrane was the best method to obtain viable cells.

#### 3.3.2. Cell Adhesion

**Figure 6:**
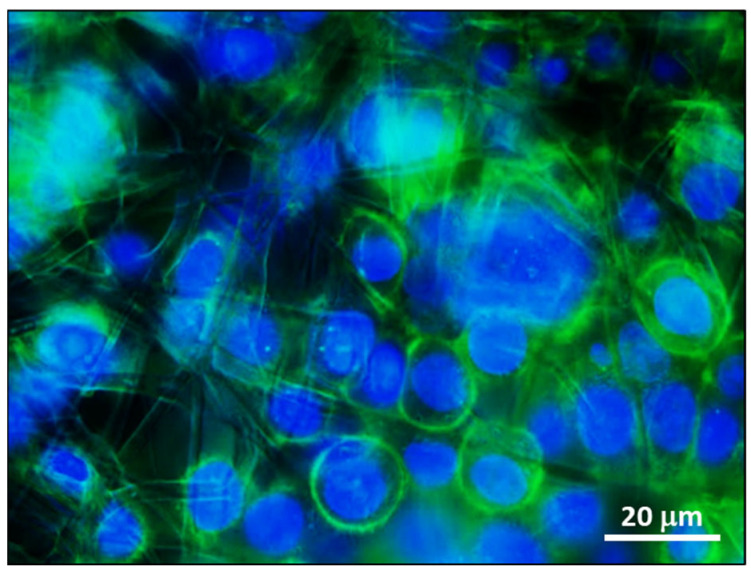
Interaction of the multilayer patch with HT29 cells. An electrospun fibers net is visible in the background. Magnification: 40×. Scale bar = 20 μm.

1-PWD electrospun-loaded patches were cut out to fit the bottom of the 24-well plate. 2.5 × 10^5^ HT29 cells were seeded on a membrane with the same method previously described. After 24 h, cell membrane dishes were transferred on glass slides, fixed, and permeabilized. F-actin was stained with Alexa Fluor 488-conjugated Phalloidin according to the manufacturer’s instructions. DAPI was added for nuclei counterstaining. Images were acquired at a magnification of 40× with a Zeiss Axio Observer inverted fluorescent microscope using FITC and DAPI filters. Our results showed that cells aggregate and adhere to the surface of the fibers, which provided a good adhesion place (Figure 6).

#### 3.3.3. Proliferation Test

To evaluate the proliferation rate of cells on the 1-PWD electrospun-loaded membrane compared to membranes alone, 250 μL of HT29 cell suspension containing, respectively, 1.5 × 10^5^ and 3 × 10^5^ cells were added onto membranes and incubated for 30 min (37 °C, 5% CO_2_). After 24, 48, and 72 h of culture in an incubator at 37 °C and 5% CO_2_ conditions, membrane dishes were transferred on glass slides, fixed, stained with crystal violet, and observed with an optic microscope.

**Figure 7:**
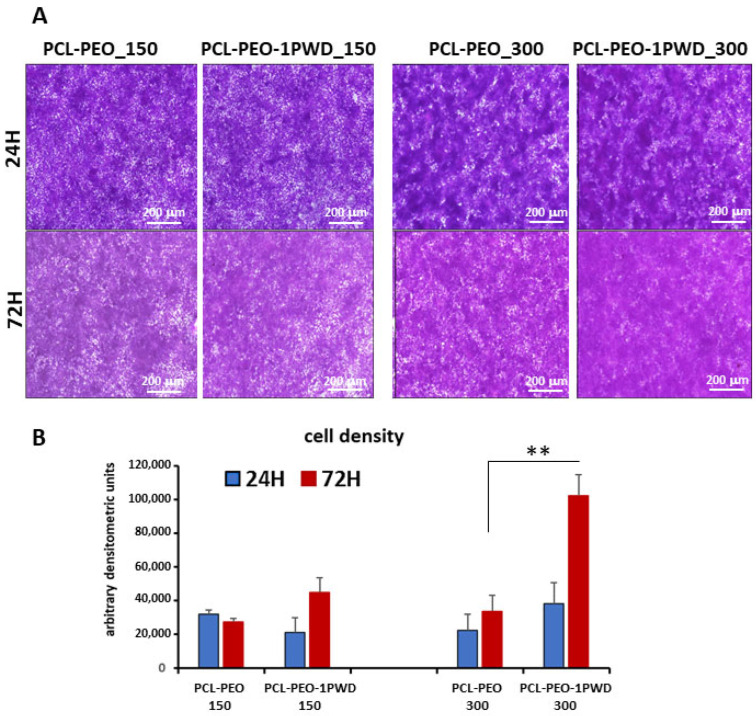
(**A**) Crystal violet staining was conducted to assess HT29 cell proliferation. Magnification: 4×; scale bar = 200 μm. (**B**) The proliferation rate of HT29 (with double cell concentration) on 1-PWD-loaded electrospun membranes vs. the control (i.e., electrospun membranes without APIs). Statistics on bars indicate differences compared to the condition W/O 1-PWD (only PCL-PEO) determined by a one-way ANOVA (** *p* < 0.01).

After 24 h of incubation, no significant differences were detected between the two conditions at both cell concentrations, while, after 72 h of incubation, a significantly denser confluent layer was observed on 1-PWD-loaded membrane membranes at the condition of 3 × 10^5^ seeded cells, suggesting a higher proliferation rate compared to membranes alone (Figure 7A). A cell growth curve was carried out after densitometric analysis of membranes by Image J software (Figure 7B).

#### 3.3.4. Cell Single Layer Migration Assay (Wound Closure Assay)

Cell migration is essential for many physiological processes, including embryonic development, wound repair, angiogenesis, and tumor metastasis [27,28]. Cell migration largely depends on the actin cytoskeleton [29], and the directional migration of fibroblasts and tumor cells depend on microtubules [30].

To investigate if 1-PWD loaded into membranes influences the migration rate of cells, we performed a scratch test on epithelial cells HT29 and the murine fibroblast cell line NIH3T3. HT29 cells and NIH3T3 cells were seeded at densities of 3 × 10^5^ and 2.5 × 10^5^, respectively, on two 24-well dishes. Membranes loaded with 1-PWD were placed at the bottom of each well. PCL-PEO-PCL membranes, without 1-PWD, were used as the negative control. Each well had special wedge inserts, which served to anchor the patch at the bottom of the well and create a “wound field”, with a defined gap of 0.9 mm, needed for measuring the migratory and proliferation rates of cells. After 24 h of incubation at 37 °C and 5% CO_2_, inserts were removed, and cells were incubated for an additional 48 h.

**Figure 8:**
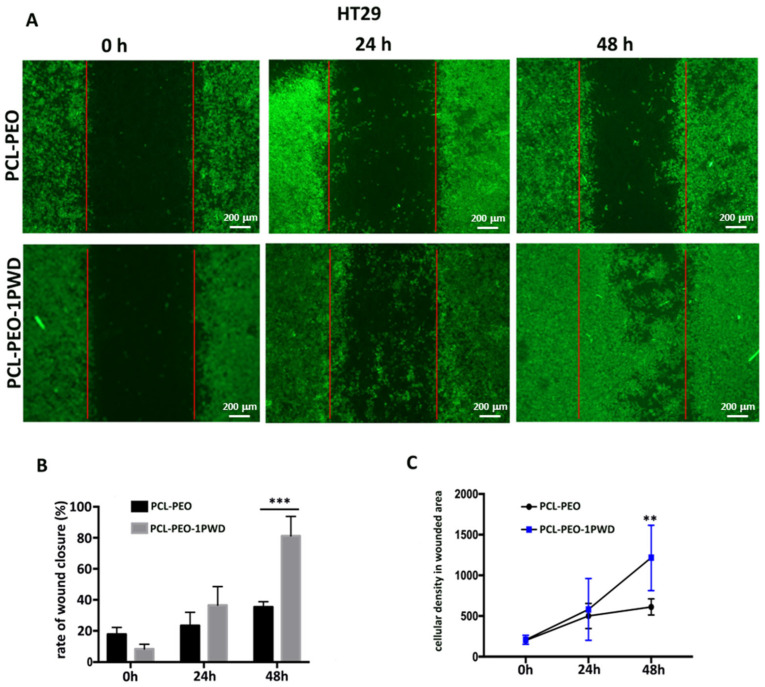
(**A**) The scratch wound test for HT29 epithelial cells on a multilayer system with and without 1-PWD. Red lines indicate the original wound edges. Magnification: 4×; scale bar = 200 μm; (**B**) the closing percentage wound values after 24–48 h of exposure. Statistics on bars indicate differences compared to the condition W/O 1-PWD (only PCL-PEO) determined by a one-way ANOVA (*** *p* < 0.001); (**C**) graph of the cellular density of the wounded area in HT29 cells on two different conditions. Statistics on bars indicate differences compared to the condition W/O 1-PWD (only PCL-PEO) determined by a one-way ANOVA (** *p* < 0.01).

**Figure 9:**
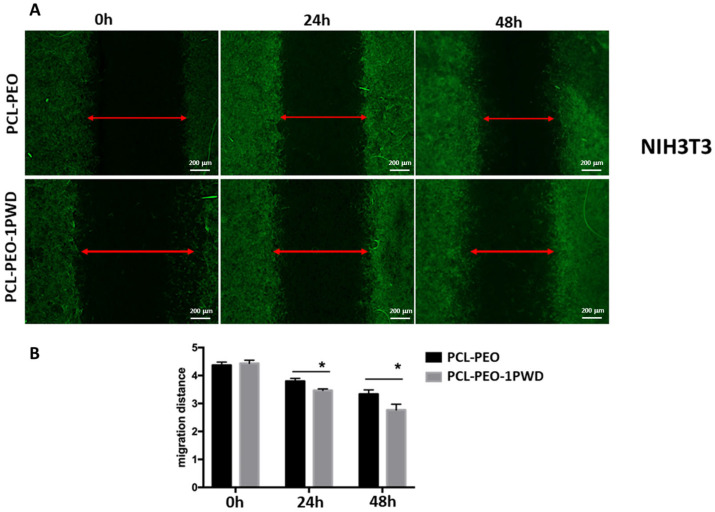
(**A**) In vitro wound healing assay. The NIH3T3 cells seeded on the surface of multilayer patches were monitored for 24–48 h. Double arrow line indicates wound width; scale bar = 200 μm; (**B**) analysis of migration distance of NIH3T3 cells on different types of membranes at 0, 24, and 48 h. Data are shown as mean ± SD (* *p* < 0.05, by one-way ANOVA).

At 24 and 48 h, cells were stained by immunofluorescence detection of F-actin, observed with a fluorescence microscope, and the gap closure was measured (Figure 8A and Figure 9A). A significant increase in cell proliferation and migration with a consequent more rapid closure of the gaps was observed in both cell lines after 48 h in cells grown on 1-PWD-loaded membranes compared to cells grown on membranes without 1-PWD (Figure 8B and Figure 9B). Remarkably, though, a prevalence of migratory cells was observed for HT29, forming dendrites of colonies protruding through the gap and eventually bridging across the wound field. Cell density of HT29 related to a fixed wounded area (1 mm^2^) was measured after 24 and 48 h using ImageJ software, as displayed in Figure 8C.

### 3.4. Controlled Release of 1-PWD© Extracts from the Electrospun System

**Figure 10:**
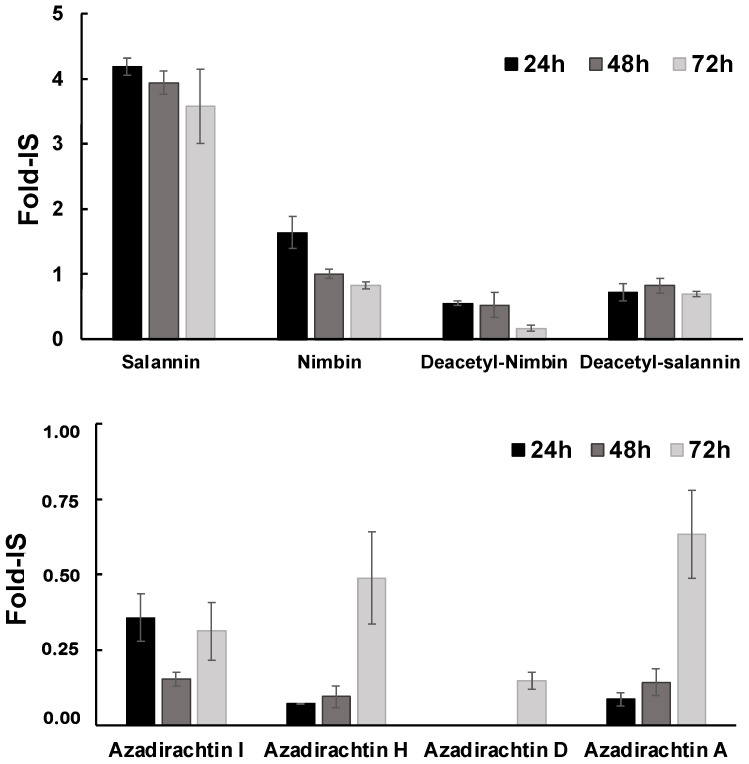
Relative values for detected metabolites expressed as folds on the level of the internal standard (Fold-IS). Data are presented as average ± standard deviation from three replicates.

To further verify the release of 1-PWD oil components from the electrospun membranes, the culture medium was analyzed by LC-HESI-HRMS after 24, 48, and 72 h of incubation with the patch loaded by 1-PWD. Recalling that 1-PWD is a well-characterized compound API made of several oily extracts (see Section 2), a number of characteristic mono-/tri-terpenoids as previously reported in the oily extract used for 1-PWD were detected, such as nimbin, salanin, deacetyl-nimbin, deacetyl-salanin, and the four forms of azadirachtin, namely I, H, D, and A [21,31,32], whose levels were measured in terms of Fold-IS (Internal Standard, Formononetin) and shown in Figure 10. In detail, salannin, nimbin, and their deacetylate forms showed an efficient release in the medium at the highest levels after 24 h, slightly decreasing during 72 h of incubation. Instead, most of the Azadirachtins (limonins) showed progressively increased levels during the incubation time, reaching the maximum values after 72 h, with azadirachtin D being detectable only after 72 h of incubation (ref. also Table 1). Even if only a subset of plant-derived oily compounds was detected and profiled in our extraction/LC-HRMS conditions (see Section 2), since most likely other compounds could be detected in more lipophilic conditions, we basically verified that these metabolites were released in the culture medium after the incubation with the 1-PWD-loaded patch.

Therefore, the LC-HESI-HRMS and biological assay results confirm that diverse oily extracts from the electrospun membranes are released during the first 72 h, even though they have different trends and are collectively capable of boosting the therapeutic efficacy of the loaded patch compared to the bare 1-PWD.

## 4. Conclusions

A functional nanofibrous wound dressing (PCL/1-PWD-PEO/PCL) was successfully prepared by loading 1-PWD in the core layer of PCL/PEO nanofibers, demonstrating that such a method of encapsulation can yield a more physiological wound healing process than bare 1-PWD. To our knowledge, this is a new methodology to engineer a multi-layer nanofibrous membrane and enable the heterogeneous loading of a combination of two natural products, neem oil and the oily extract of Hypericum perforatum flowers. The obtained composite prototype holds considerable potential for wound healing applications. The use of a membrane capable of the controlled release of natural oils could indeed offer numerous advantages compared to the application of free oils, such as a reduction in the frequency of wound dressing applications, which limits the infection risk, the use of antibiotics, and the pain associated with dressing replacement.

The characterization by SEM, contact angle measurement, FTIR, and LC-HRMS highlighted key aspects of these electrospun patches. According to SEM, the loading of 1-PWD does not significantly alter the fiber distribution of the parent (unloaded) electrospun construct, apart from a small increase in the average fiber diameter, which is in qualitative agreement with other studies about electrospun fibers added with essential oils [33,34]. A facile route to detect the loading of the 1-PWD into the PCL-PEO nanofiber mats was obtained by FTIR spectra; however, in-depth drug-release studies by LC-HRMS rendered the ultimate proof of the loading of our API and its sustained release after 72 h in a physiological fluidic environment, albeit with different release kinetics for each component of 1-PWD. In addition, wettability results showed that the PCL layer can absorb the 1-PWD oily mix, thus providing a possible mechanism to re-load a patch with a fresh API, e.g., to refill an exhausted patch and further tweak or extend the release profile.

The biological validation by in vitro cell culture experiments for nanotoxicity demonstrated that the incorporation of 1-PWD into the electrospun PCL-PEO-PCL layered fiber mat yielded no adverse effects on cell viability. More importantly, the 1-PWD-loaded patch showed excellent biocompatibility toward HT29 and NIH3T3 cells, as well as an optimal in vitro wound healing efficacy proven by proliferation and scratch assays.

All these results suggest that the proposed 1-PWD patch concept is a potential candidate for next-generation wound healing dressing in the context of the green economy, leveraging the action of natural API therapeutic compounds that can be also refilled. Future studies could include the actual pre-clinical validation of this medical device through selected in vivo animal models.

## Figures and Tables

**Table 1:** Values for detected metabolites are expressed as folds relative to the level of the internal standard. Data are presented as the average ± standard deviation from three replicates.

Metabolite	24 h	48 h	72 h
Salannin	4.19 ± 0.133	3.94 ± 0.18	3.57 ± 0.568
Nimbin	1.64 ± 0.241	1.00 ± 0.072	0.82 ± 0.052
Deacetyl-Nimbin	0.55 ± 0.031	0.53 ± 0.187	0.17 ± 0.049
Deacetyl-Salannin	0.72 ± 0.132	0.82 ± 0.108	0.69 ± 0.039
Azadirachtin I	0.36 ± 0.079	0.15 ± 0.024	0.31 ± 0.096
Azadirachtin H	0.07 ± 0.002	0.09 ± 0.035	0.49 ± 0.152
Azadirachtin D	-	-	0.15 ± 0.029
Azadirachtin A	0.09 ± 0.021	0.143 ± 0.044	0.63 ± 0.146

## Data Availability

Data are available from the corresponding author upon reasonable request.
